# Supplementary material for: Socio-economic factors associated with adolescent pregnancy and motherhood: Analysis of the 2017 Ghana maternal health survey
Source: PLoS One. 2022 Dec 30;17(12):e0272131. doi: 10.1371/journal.pone.0272131 (PMC9803283; doi:10.1371/journal.pone.0272131)
Supplement: S1 File — (PDF) [file pone.0272131.s001.pdf]

References

19. Bekwa BG. Life experiences of multiparous teenage mothers in selected communities in the Eastern Cape. M.Sc. Thesis, University of South Africa. 2020. Available from: https://uir.unisa.ac.za/handle/10500/26773

24. Okine L. Exploring the experiences of teenage mothers with repeat pregnancies in Ablekuma sub-metropolis. M.Sc. Thesis, University of Ghana. 2019. Available from: http://ugspace.ug.edu.gh/handle/123456789/29346

28. Dlamin N. Teenage mothers' experiences of motherhood- schooling, stigma and learned responsibility: a case study of teenage mothers of school going age in a peri-urban area in Kwa-Zulu Natal. M.Sc. Thesis, Stellenbosch University. 2016. Available from: http://hdl.handle.net/10019.1/98667

36. Babae PMN. Factors influencing adolescent pregnancy in the Upper East region of Ghana . M. Sc. Thesis, Royal tropical institute. 2017. Available from: https://bibalex.org/baifa/en/resources/document/476516

55. Asamoah J. Causes of teenage pregnancy in Senya Bereku community of Awutu- Effutu-Senya district. M.Sc. Thesis, University of Ghana. 2005. Available from: http://ugspace.ug.edu.gh/bitstream/handle/123456789/6345/

64. Mamboreo JN. Factors influencing age at first birth in coast provinces. M.Sc. Thesis, University of Kenya. 2012. Available from: http://erepository.uonbi.ac.ke/handle/11295/24493

66. Baafi D. Adolescent pregnancy in an urban community: a study in the Sunyani municipality. M.Sc. Thesis, University of Ghana. 2015. Available from: http://ugspace.ug.edu.gh/handle/123456789/8850

82. Cantet N. The effects of teenage pregnancy on schooling and labour force participation: evidence from urban South Africa. PhD Thesis, University of Cape Town. 2019. Available from: https://cpb-us-w2.wpmucdn.com/sites.wustl.edu/dist/e/763/files/formidable/11/NataliaCantet.pdf
